# Transcranial pulse stimulation modulates neuronal activity and functional network dynamics

**DOI:** 10.1016/j.brs.2025.09.021

**Published:** 2025-09-29

**Authors:** Maria Eleni Karakatsani, Irmak Gezginer, Daniil Nozdriukhin, Savannah Tiemann, Hikari A.I. Yoshihara, Rafael Storz, Markus Belau, Ruiqing Ni, Xosé Luís Deán-Ben, Daniel Razansky

**Affiliations:** a Institute for Biomedical Engineering and Institute of Pharmacology and Toxicology, Faculty of Medicine, University of Zurich, Winterthurerstrasse 190, CH-8057, Zurich, Switzerland; b Institute for Biomedical Engineering, Department of Information Technology and Electrical Engineering, ETH Zurich, Wolfgang-Pauli-Strasse 27, CH-8093, Zurich, Switzerland; c Institute for Regenerative Medicine, Wagistrasse 12, 9th floor, CH-8952, Zurich, Switzerland; d Storz Medical AG, Lohstampfestrasse 8, CH-8274, Tagerwilen, Switzerland

**Keywords:** Transcranial pulse stimulation, Therapeutic ultrasound, Calcium influx, Reorganization of functional network

## Abstract

**Background:**

Transcranial pulse stimulation (TPS) has recently been proposed as a promising non-invasive technique for treating neurological disorders. While neuropsychological improvements in treated Alzheimer’s disease (AD) patients support its safety and preliminary clinical effectiveness, the fundamental mechanisms of TPS action on the brain remain unclear.

**Objective:**

In this study, we explore the effects of TPS on neuronal activity and brain circuitry in healthy and AD mouse models.

**Methods:**

We utilized fluorescence calcium imaging combined with resting-state functional magnetic resonance imaging and c-Fos immunohistochemistry for validation.

We imaged TPS-treated mouse brains expressing genetically encoded calcium indicator and compared the imaging data from AD mouse strains to wild-type controls, followed by immunohistochemical analysis of neuronal activation to support the *in vivo* imaging findings.

**Results:**

TPS induced robust calcium influxes in GCaMP + mice, increased c-Fos expression in the dentate gyrus, and rapidly but transiently reorganized functional connectivity across brain networks, particularly within the hypothalamus, hippocampus, and other limbic regions. At higher stimulation intensities, TPS is shown to trigger spreading depolarization waves.

**Conclusion:**

These findings support the hypothesis that TPS-induced mechanical effects can effectively modulate brain activity while averting tissue heating and cavitation, thus shedding light on observed beneficial effects in patients and paving the way for further optimization of TPS as a therapeutic strategy for neurodegenerative diseases.

## 1. Introduction

Medical applications of ultrasound (US) in neurology have advanced considerably over the past decades, enabling ablation of pathological brain tissue, drug delivery via blood-brain barrier (BBB) opening, and neuromodulation [1,2]. Technological and scientific advances have facilitated transcranial US propagation, precise beam targeting to mm-sized focal areas, and non-invasive stimulation [1]. Preclinical investigations have revealed important mechanisms underlying US application under different thermal, mechanical, or cavitational forces driving downstream neurological responses [3]. Clinical applications have recently surged, offering promising therapeutic avenues to patients with no other effective alternatives [4]. With the rising life expectancy, the aging population is progressively suffering from neurodegenerative disorders, which are known to involve inherently complex, long-term, and multi-factorial processes. Effective medications eliminating brain pathology and ameliorating the progressing dementia are often unavailable, while invasive measures are generally not practical due to the patients’ age. As such, US brain stimulation has been proposed as a promising therapeutic strategy benefiting from a non-invasive approach that can reach deep brain regions to trigger signaling pathways promoting survival [1].

Several studies have been conducted to explore the potential mechanisms behind the observed neuromodulatory effects of US. Neuronal membrane deformation is widely regarded as a key underlying mechanism, which triggers the activation of mechanosensitive ion channels [5–7] and eventually leads to membrane depolarization and signal transduction. In particular, US can directly drive a number of mechanosensitive ion channels including the K^+^ channel family [5], voltage gated Na^+^ and Ca^2+^ channels [8], and piezo-type [6,7] channels. Also, calcium responses have been observed in neurons when exposed to mechanical stress [9]. Cavitational and thermal effects have also been reported [10,11] for US exposure with particularly high pulse repetition frequency, high duty cycle, or high pressure. Under these conditions, US may increase temperature and alter the electrical capacitance of the plasma membrane [12]. Efforts have been made to disentangle a potential interaction between mechano- and thermo-sensitive ion channels. For example, knocking out the mechanosensitive channels silenced neuronal responses to stimulation, while knocking out the thermosensitive channels did not have an effect [13]. Overall, depending on the pulse regime, different combinations of partially overlapping mechanisms could contribute to the interaction between US and the cell membrane [14].

Unlike other US-based techniques, TPS utilizes transcranially applied ultrashort (around 3 μs duration) pressure impulses with a pulse repetition frequency between 1 and 8 Hz and per-pulse energy density of up to 0.25 mJ/mm^2^ in the treated focal region [15]. This mainly results in mechanical responses while averting tissue heating. A TPS medical device has been approved for Alzheimer’s disease (AD) therapy under Conformité Européene (CE) certification and received Investigational Device Exemption (IDE) approval from the United States Food and Drug Administration (FDA). The method has shown remarkable effectiveness in treating AD, with a number of clinical studies showing improved neuropsychological scores lasting up to three months, upregulation of memory networks, and anti-depressive effects [16–18]. In our recent preclinical study, no evidence of BBB opening, fluid extravasation or other detrimental effects to the brain resulting from TPS procedures were found [19]. Despite its robust safety profile and preliminary evidence of clinical effectiveness, the fundamental modes of TPS action on the brain remain poorly understood.

In this work, we systematically investigate the neuromodulatory effects of TPS in mice, anticipating the activation of mechanosensitive ion channels to lead to membrane depolarization and calcium channel activation as well as upregulation of brain networks associated with memory circuits *in vivo*. These hypotheses are based on preclinical findings by the broader brain stimulation community, which were yet made under different parametric regime of US application [14]. We performed real-time *in vivo* wide-field fluorescent (FL) calcium imaging of TPS-treated mouse brains expressing genetically-encoded calcium indicator (GCaMP) in combination with resting state functional magnetic resonance imaging (rs-fMRI). The imaging data from AD mouse strains was compared to wild-type (WT) controls, followed by immunohistochemical analysis of neuronal activation to support the *in vivo* imaging findings.

## 2. Materials and methods

### 2.1. Study design

The objective of the current study is: i. to detect any neuronal activity during TPS, ii. identify the spatial distribution of activated neurons with immunohistochemistry, and iii. report any functional network synchrony reorganization. To perform these tasks various animal models were included, each providing complementary information to the study.

The animals in this study were housed in ventilated cages under controlled conditions (21 ± 1 °C, 55 ± 10 % humidity) with a 12-h light/dark cycle. Pellet food and water were provided *ad libitum*. All experiments complied with the Swiss Federal Act on Animal Protection and were approved by the Cantonal Veterinary Office Zürich. Three mouse strains were employed in order to assess neural activity induced by TPS, namely GCaMP+ (N = 3, 8–12 weeks old male, The Jackson Laboratory), GCaMP− (N = 3, 8–12 weeks old male, The Jackson Laboratory), CD1 (N = 6, 8–12 weeks old male and female, Charles River laboratories), and 5xFAD mice (N = 6, 8–12 weeks old male, The Jackson Laboratory). Two mouse strains were used to assess functional network dynamics, namely B6129SF2/J (N = 8, 50–55 weeks old male and female, The Jackson Laboratory) and 3xTg-AD (N = 8, 50–55 weeks old male and female, B6; 129-Tg(APPSwe, tauP301L)1Lfa Psen1tm1Mpm/Mmjax, The Jackson Laboratory). GCaMP + mice express this genetically encoded calcium indicator in neuronal cells, exhibiting increased FL following calcium binding. 5xFAD harbor five mutations leading to overexpression of mutant human amyloid beta protein, and 3xTg-AD mice contain three mutations associated with familial AD resulting in progressive neuropathology including plaques and tangles. GCaMP−, CD1, and B6129SF2/J served as age-matched WT strains. Animals were randomly assigned in equal sample size groups when possible. The baseline FL recording served as the “sham” acquisition for every GCaMP animal individually, therefore they all received TPS subsequently.

### 2.2. Transcranial pulse stimulation (TPS)

TPS was applied using a clinically approved handpiece (Neurolith, Storz Medical AG, Switzerland) used in human applications. It delivers ultrashort impulses with a duration of approximately 3 μs. The elongated focal area encompassed the entire mouse brain with the highest ultrasound intensity concentrated in the lateral center [19] and was oriented normally to the mouse brain surface with the axial focal position located at the surface. The effective beam size has a full width at half maximum (FWHM) of 56 mm in the axial direction and 5.3 mm in the lateral direction. The pulses were emitted at a frequency of 4 Hz for a total of 100 pulses, with per-pulse energy densities at the focus of 0.05 mJ/mm^2^ (“low energy”) and 0.25 mJ/mm^2^ (“high energy”). The low energy level was selected to approximate typical focal energy levels used in a clinical setting while accounting for differences in skull thickness and attenuation between humans and mice. The highest energy level was then used to tentatively induce stronger neuromodulatory effects. The stimulation paradigm consisted of 3 bursts each containing 100 pulses (low, low, high energy – 300 pulses in total) separated by 1-min intervals. Application of the same 3 bursts was repeated after 10–15 min.

### 2.3. Epifluorescence imaging

**Fig. 1.**
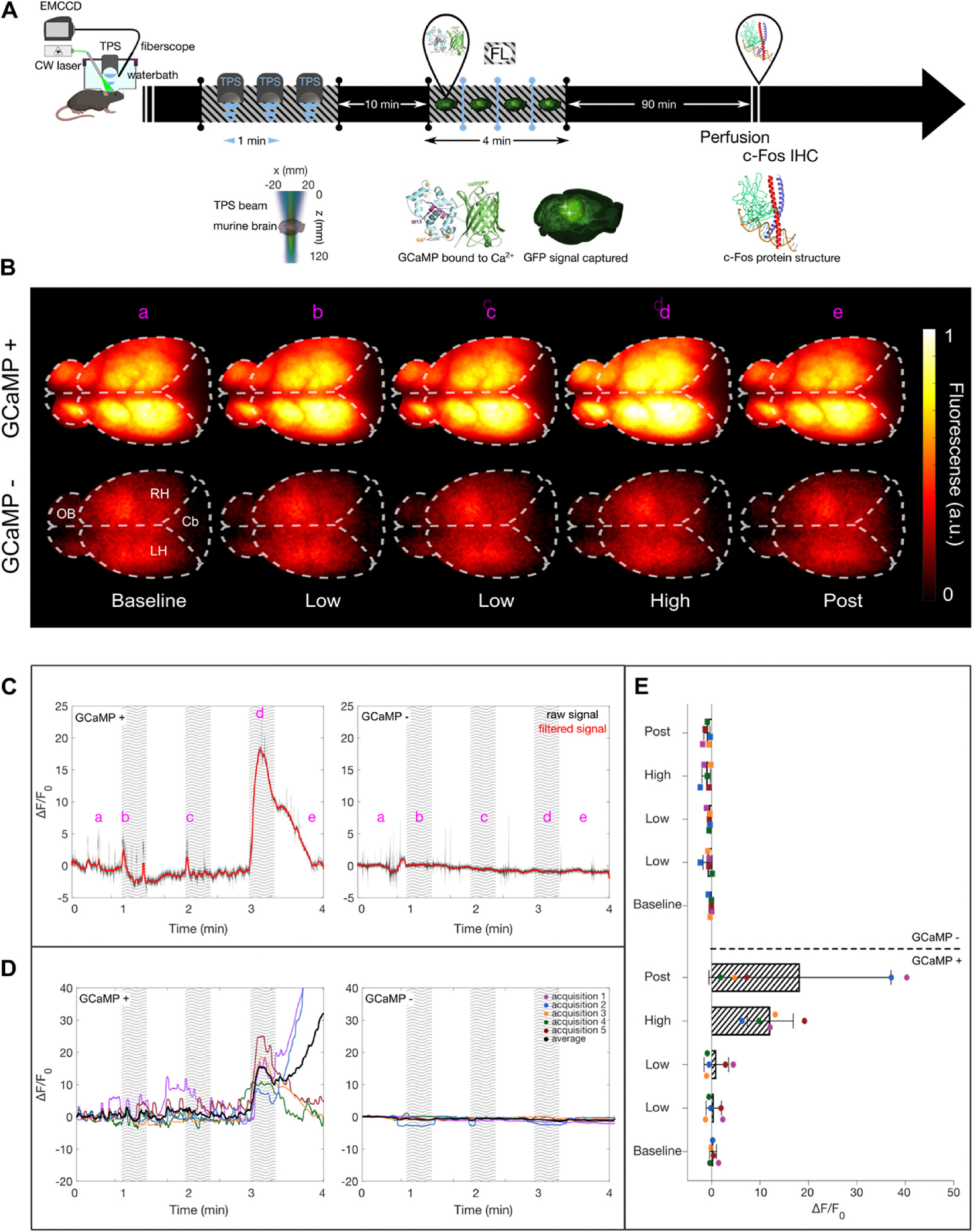
Fluorescent calcium imaging reveals neuronal responses to TPS. (A) Pipeline of the stimulation during fluorescent imaging. EMCCD: Electron-multiplying CCD camera; GFP: green fluorescent protein; IHC: immunohistochemistry (**B)** Representative examples of a GCaMP positive (GCaMP+) and a GCaMP negative (GCaMP−) animal and brain images from the experimental intervals: baseline (prior to stimulation), low (stimulation at the low energy setting), low (second stimulation at the low energy setting), high (stimulation at the high energy setting), post (following the stimulation). The fluorescence signal intensity increases during TPS and correlates with TPS energy level. (**C)** Quantification of the relative increase in the fluorescing signal (ΔF/F_0_). Signal increase during high-intensity stimulation, with a subsequent return to baseline, is evident for the GCaMP + brains while no apparent changes are observed for the GCaMP− brains. (**D)** All successful acquisitions from GCaMP+ and GCaMP− animals have coherent response patterns across most intervals. Despite the similarity in the signal pattern until the high energy TPS interval, in two out of the five cases, signal saturation followed instead of a return to baseline (Movies S3, S4). (**E)** The average signal intensity in the high energy TPS interval is, as expected, significantly increased compared to all other intervals, proven by two-way ANOVA with Holm-Šídák correction for multiple comparisons.

FL responses from the brains of GCaMP mice were recorded during TPS stimulation using a flexible fiberscope (Zibra Corporation, Westport, USA) in epi-illumination mode with a pixel resolution of ~40 μm [20,21]. The fiberscope features a 1.4 mm diameter optical image guide composed of 100,000 fibers. It was positioned at a 35-degree angle relative to the TPS handpiece, which was oriented perpendicularly to the mouse brain surface and placed approximately 40 mm away from it to ensure the focal volume of the handpiece to cover the brain hemisphere (Fig. 1A). FL excitation was achieved using a continuous-wave (CW) 488 nm laser at 50 mW power (Sapphire LPX 488–500, Coherent, USA), which was coupled to a custom-made fiber bundle (Lightguide GmbH, Germany) with a 5 mm core diameter and containing approximately 500 fibers, each with a numerical aperture (NA) of 0.27. The bundle was oriented at 35° relative to the TPS handpiece at the opposite side of the fiberscope. FL responses were captured at 20 fps with 50 ms exposure time using a cooled EMCCD camera (iXon Life 888, Andor, UK) coupled to the fiberscope.

Three GCaMP+ and three GCaMP− animals were initially anesthetized with an air-oxygen mixture and isoflurane (100–400 mL/min, 5 % v/v for induction and 2 % v/v for maintenance, Abbott, Cham, Switzerland). The animals were fixed into a custom-designed stereotactic holder coupled to a breathing mask on a heated bed. The top of the head was depilated and covered with US gel to ensure proper TPS coupling. The animals were continuously monitored with FL imaging for 8 min with a 10-min break halfway. Within this time frame the animals received two TPS applications with three bursts each. The duration from induction to completion of the TPS application was 30 min. All animals were transcardially perfused 90 min later (30 mL PBS followed by 30 mL 4 % paraformaldehyde (PFA) in PBS) to allow adequate time for c-Fos expression [22]. The brains were extracted, preserved in PFA for 24 h and then transferred to 30 % sucrose for at least 2 days prior to freezing, sectioning, and immunohistochemistry. Due to the limited size of the GCaMP + cohort and since baseline frames (>2000) were acquired to control the FL imaging session, only one GCaMP + animal served as a sham that underwent the entire experimental procedure without TPS stimulation.

### 2.4. FL image processing

The entire FL image acquisition consisted of 10,000 frames each corresponding to a 50 ms exposure time. The 2D images were processed to remove the background and retain the FL signal. The first 500 frames were omitted to remove initial emission fluctuations. For the remaining frames, the median signal intensity was calculated, to produce a signal time series, that was smoothened with a 100-order 1D median filter. The relative increase in the FL signal (ΔF/F_0_) was computed by the percentile change of the signal at each time point compared to the baseline signal (average signal of the frames 101:2000). The raw and filtered relative changes are shown in Fig. 1C. For statistical analysis, the relative signal change was averaged over the pulses corresponding to the same interval; low energy level frames: 2400–3400, second low energy level frames: 4800–5800, high energy level frames: 7200–8200, post TPS frames: 8201:10000.

### 2.5. Immunohistochemistry and microscopy

The GCaMP, CD1 and 5xFAD brains were cut in coronal hippocampal sections to a thickness of 35 μm using a microtome (CM3050S, Leica Biosystems, Germany). 5–8 free-floating sections with a 6-sections gap were first washed in 0.1 M PBS and blocked in a solution of 5 % donkey serum in 0.3 % PBS with Triton X-100 (PBST) for 30 min. The tissue was incubated overnight at 4 °C with the primary rabbit *anti*-c-Fos antibody (1:1000 226,008, SYSY antibodies). On the second day, sections were washed in 0.3 % PBST and then incubated for 60 min in a solution of 5 % donkey serum in 0.3 % PBST containing donkey-raised secondary Alexa Fluor 647 antibody (1:1000, A-31573, Invitrogen). The tissue was then washed with 0.1 M PBS and mounted on slides. After allowing the tissue to dry, the slides were cover-slipped with Dako Fluorescence Mounting Medium (S302380–2, Agilent).

Large field confocal images of the hippocampus were captured on a Zeiss LSM 900 AiryScan 2 confocal microscope (Carl Zeiss AG, Oberkochen, Germany) with the same laser exposure parameters for every slide. A 10× objective with 0.3 NA was employed, corresponding to 5.2 mm working distance and 1.1 μm resolution at the 520 nm excitation wavelength. Tile (mosaic) and a Z-stack (1 μm step, 5 series) were obtained and processed to produce maximum intensity projection (MIP) images.

### 2.6. c-Fos image processing

**Fig. 2.**
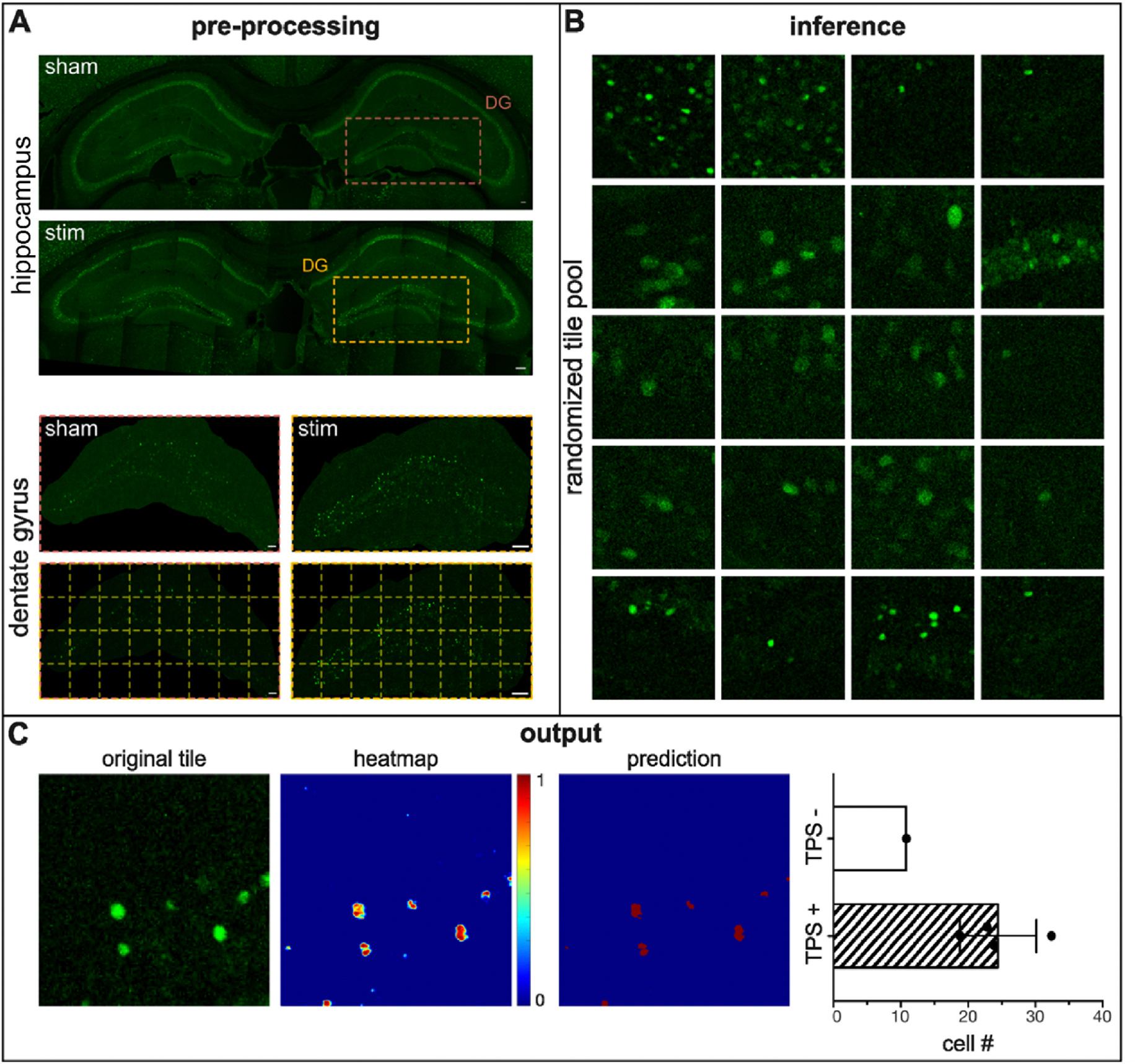
Automated c-Fos quantification shows increased activity following TPS. (A) Microscopy images covering the entire hippocampus were acquired and preprocessed to isolate the DG, remove the background and normalize their intensity. The DG images were split into tiles and brought into the proper matrix size 1200×1200 to feed into the CNN. (**B)** The tiles from all the GCaMP brain images were analyzed with a pre-trained CNN model in random order. Part of the randomized tile pool is shown herein. (**C)** The model’s output is a heatmap with the probability of a pixel corresponding to a cell. Pixels exceeding the probability of 0.7, are considered part of a cell. The detected cells from the tiles compromising one image, were summed and all brain sections corresponding to the same brain averaged and plotted. The brains that received TPS showed more than a two-fold increase in the c-Fos positive cell count compared to the sham brains.

The c-Fos processing pipeline was entirely implemented in Python 3.7, including the pre-processing and the inference steps shown in Fig. 2A and B. Initially. the MIP image of the hippocampus was pre-processed to isolate the dentate gyrus (DG) and remove the background noise. Background removal involved a Gaussian blurring with a 5×5 kernel followed by an edge detection filter. The filtered image of the DG was split into tiles at the 128×128 matrix size. Each tile was processed with a morphological opening, using a disk-shaped structuring element with a radius of 1 pixel, to remove small objects. The tile was then interpolated to reach the matrix size of 1200×1200. Image splitting and tile resizing were dictated by the pre-trained network and the corresponding training dataset.

The tiles from all available images per strain (GCaMP: 31 images, 3941 tiles; CD1: 73 images, 17081 tiles; 5xFAD: 28 images, 2334 tiles) were randomized and fed into a pre-trained fully convolutional neural network (CNN) [23]. In brief, the issue of automated cell counting in FL microscopy has been addressed with a deep-learning approach [23]. For this, a fluorescent neuronal cells dataset consisting of 283 high-resolution images with ground truth labels was used and tested with four different CNNs [23]. According to the evaluated metrics, detection performance, and accurate counts, the cell ResUnet (c-ResUnet), boosted with weight maps and crowded areas, was found to prevail among the four networks [23]. The output of the network is a probability map whereas each pixel value corresponds to the probability belonging to a cell (Fig. 2C).

In the post-processing step, we set a threshold of 0.7 for the model prediction, which is the cutoff probability of a pixel belonging to a cell. Cells were finally counted by means of connected components and the total number within a tile was recorded. The detected cells from the tiles comprising one image were summed and all brain sections corresponding to the same brain were averaged and plotted (Fig. 2C).

### 2.7. In vivo MRI

Both wild-type (B6129SF2/J, N = 8) and transgenic (3xTg-AD, N = 8) mice underwent similar fMRI procedures. Anesthesia was induced with 4 % isoflurane in air-oxygen mixture and maintained at <1.5 % during initial preparations (head fixation, magnetic field homogeneity adjustments, shimming, and reference pulse acquisition). Subsequently, mice received an intravenous (i.v.) bolus of medetomidine (0.05 mg/kg; Domitor, medetomidine hydrochloride, Pfizer Pharmaceuticals, Sandwich, UK). Five minutes after the bolus, isoflurane was reduced to 0.7 % to facilitate sedation. A continuous infusion of medetomidine (0.1 mg/kg/h) was initiated 10 min after the bolus to maintain stable sedation. fMRI data acquisition began 40 min following medetomidine administration after an anatomical MRI scan had been completed.

Each functional imaging session consisted of three 15-min acquisitions. The first scan served as the baseline measurement. For the TPS experiments (N = 12; 6 B6129SF2/J and 6 3xTg-AD), mice were carefully removed from the scanner without altering their orientation on the animal bed, and underwent the first TPS (low, low, high energy; see TPS section). They were then repositioned in the scanner, and the second functional acquisition (Post-TPS 1) began approximately 2 min later (range: 1 min 49 s–2 min 21 s post-stimulation). Following TPS 1, a second TPS (TPS 2) was administered under the same parameters, after which the third 15-min functional acquisition (Post-TPS 2) was performed. Throughout the session, respiratory rate and rectal temperature were continuously monitored, and body temperature was maintained at 37 °C using an MR-compatible, feedback-controlled water heating system.

For the sham experiments (N = 4; 2 B6129SF2/J and 2 3xTg-AD), all procedures were identical to the TPS stimulation protocol except that no actual stimulation was delivered. Mice designated for sham procedures were removed from the scanner, had gel applied to their scalps, and the transducer positioned on their heads for the same duration as the real stimulation but without actual TPS emission. They were then returned to the scanner for the subsequent acquisitions. As with the TPS stimulation experiments, three 15-min functional scans were acquired, with the sham procedures interleaved between the scans.

### 2.8. fMRI data acquisition

All MRI experiments were performed on a 7.0 T MRI system (Biospec 70/16, Bruker BioSpin, Ettlingen, Germany) equipped with a cryogenic quadrature RF surface probe (CryoProbe, Bruker BioSpin AG, Fällanden, Switzerland). Anatomical images were acquired using a T2-weighted rapid acquisition with relaxation enhancement (RARE) sequence with the following parameters: field of view (FOV) = 20 × 10 mm^2^, matrix size = 256 × 128, slice thickness = 0.4 mm, repetition time (TR) = 3000 ms, echo time (TE) = 15 ms, RARE factor = 8, and number of averages (NA) = 6. Functional images were obtained using a gradient echo-echo planar imaging (GE-EPI) sequence with an FOV of 20 × 10 mm^2^, matrix size of 90 × 50, in-plane voxel size of 222 × 200 μm^2^, slice thickness of 0.4 mm, flip angle (FA) = 60°, TR = 1000 ms, TE = 15 ms, and NA = 1. Prior to fMRI, B0 field maps were acquired to optimize local field homogeneity.

### 2.9. fMRI data pre-processing

Functional MRI datasets were preprocessed using SPM12 (Wellcome Trust Centre for Neuroimaging, London, UK), the CONN toolbox [24], and custom MATLAB scripts. Each subject’s anatomical and functional images were co-registered and subsequently normalized to the Allen Mouse Brain Common Coordinate Framework (CCF) [25] using both linear and nonlinear transformations. The functional images were resampled to an isotropic voxel size of 0.2 × 0.2 × 0.2 mm^3^, realigned for motion correction, and spatially smoothed with a 0.6 mm full-width-at-half-maximum (FWHM) Gaussian kernel. Temporal pre-processing included detrending and bandpass filtering in the 0.008–0.1 Hz frequency range. To minimize non-neuronal signal contributions, signals from white matter, cerebrospinal fluid, and six motion parameters (three translations, three rotations) were regressed out [26]. Finally, the preprocessed data were segmented into 5-min intervals for each acquisition for subsequent analyses.

### 2.10. Functional connectivity (FC) analysis

**Fig. 3.**
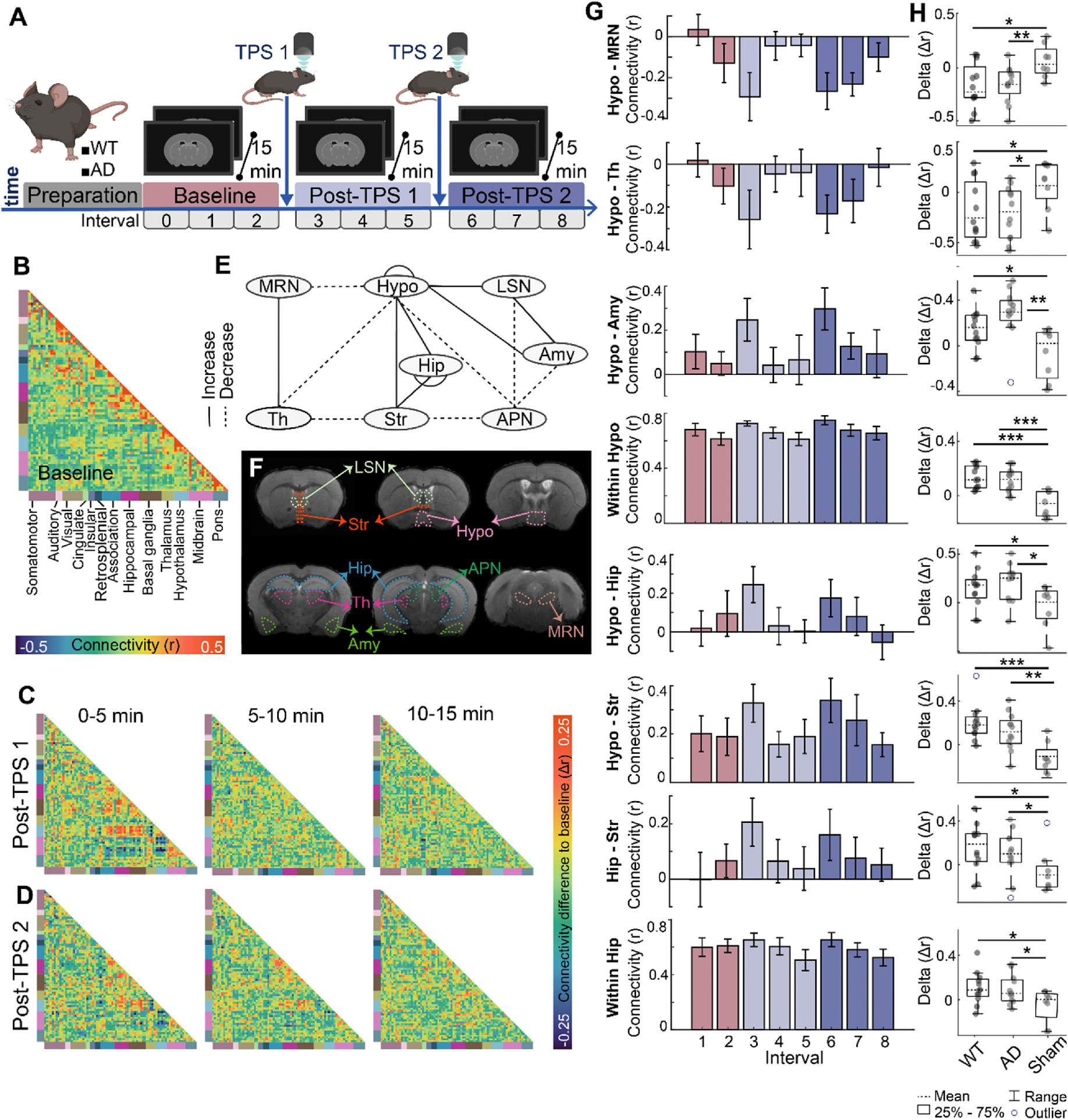
TPS-induced fMRI-based functional connectivity changes. (A) Experimental timeline. Wild-type (WT) and Alzheimer’s disease (AD) mice underwent a baseline resting-state fMRI scan, followed by two TPS stimulations, interleaved with post-stimulation scans. Scans were divided into 5-min intervals. (**B)** Group-averaged baseline FC. Functional networks are color-coded representing distinct brain regions. (**C-D)** Temporal dynamics of FC changes following TPS 1 (c) and TPS 2 (d). Differences from baseline FC are displayed for each 5-min interval. (**E)** Network diagram depicting brain regions that exhibited significant FC alterations in response to TPS. (**F)** The regions shown in (e) overlaid on an anatomical brain image. (**G)** Group-averaged FC values (mean ± SEM) for each time interval, illustrating the dynamics of region-wise connectivities. (**H)** Magnitude of FC changes compared across both stimulation cycles for WT, AD, and sham groups. (*) p < 0.05, (**) p < 0.01, (***) p < 0.001; two-sample *t*-test. MRN, midbrain reticular nucleus; Hypo, anterior hypothalamic area; LSN, lateral septal nucleus; Hip, hippocampus; Amy, lateral amygdalar nucleus; Th, lateral dorsal thalamus; Str, Striatum; APN, anterior pretectal nucleus.

FC analysis was conducted on denoised time-series data using regions of interest (ROIs) defined by the Allen Common Coordinate Framework (see Table S1). For each functional scan (Baseline, Post-TPS 1, or Post-TPS 2), the fMRI data were segmented into three consecutive 5-min intervals. Notably, the first interval of the baseline scan was excluded from further analysis due to unstable connectivity patterns, likely reflecting the animal’s acclimatization to anesthesia. Mean time series were extracted from each ROI, and pairwise Pearson’s correlation coefficients (r) were computed to generate ROI-to-ROI connectivity matrices (Fig. 3B and C). To quantify stimulation-related changes, the connectivity in the first 5-min interval following stimulation (interval 3 and 6, Fig. 3A) was compared to the average connectivity derived from the last 5-min interval of the preceding scan and the second 5-min interval of that same scan. Prior to statistical testing, correlation values were Fisher’s z-transformed. Student’s t-tests with False Discovery Rate (FDR) correction (p < 0.05) were then used to identify significant differences in connectivity, which were visualized as bar plots (Fig. 3G). The same analysis pipeline was applied to both the TPS stimulation and sham experiments. Changes in Pearson’s correlation values (Δr) in response to stimulation were plotted separately for WT animals, AD animals receiving TPS, and the sham group (Fig. 3H).

### 2.11. Statistical analysis

The statistical analysis performed in each of the quantitative endpoints have been described in the corresponding Results’ section. Processing steps are elaborately described in the materials and methods section to allow the reader a deeper understanding of the analysis.

## 3. Results

### 3.1. Fluorescent imaging reveals calcium response to TPS

The wide-field FL calcium recordings were captured prior, during and after the TPS sessions using a custom-made fiberscope system operated in epi-fluorescence mode (see Materials and Methods for details). Stimulation intervals occurred every minute and lasted for almost 25 s, while intervals of TPS inactivity were also recorded (Fig. 1A). Representative results from GCaMP positive (GCaMP+, N = 3) and GCaMP negative (GCaMP−, N = 3) animals for two stimulation intervals are shown in Fig. 1B, revealing a clear FL signal intensity increase during TPS. Quantification of the relative increase in the FL signal intensity (ΔF/F_0_) showed correlation with the TPS energy level (Fig. 1C). As expected, no apparent signal changes were evident in the GCaMP− mouse brains (Movies S1, S2). Consistent response patterns were observed across most intervals from GCaMP+ and GCaMP− animals (Fig. 1D). Despite the similarity in the signal patterns prior to the high energy TPS interval, signal saturation has been observed in two out of the five studied cases instead of an expected return to baseline (Movies S3, S4). This saturation likely reflects spreading depolarization, where large neuronal populations depolarize simultaneously, producing a strong cumulative GCaMP response that can exceed the dynamic range of the sensor. The observed saturation pattern begins from a local area of high stimulation intensity, and expands into the entire hemisphere as a propagating wave, indicative of membrane depolarization (Movies S3, S4). The average signal intensity in the high energy TPS interval is, as expected, significantly increased compared to all other intervals, as verified by two-way ANOVA with Holm-Šídák correction for multiple comparisons (Fig. 1E). These results clearly show the importance of properly adjusting the stimulation parameters (energy, intervals, duration) to modulate neuronal depolarization effects. Despite the small pilot sample size (N = 3), the TPS-induced calcium responses were highly robust and reproducible across all animals, with very large effect sizes. Blinding procedures were applied during image analysis.

### 3.2. Increased c-Fos activity following TPS

Immunohistochemical analysis against the c-Fos protein showed increased protein levels, following TPS compared to the inherent brain levels, in the DG subfield of the hippocampal formation, which is critical for spatial memory formation [27]. According to the experimental timeline (Fig. 1A), the animals were transcardially perfused 90 min after the last TPS session to allow expression of the c-Fos gene [22]. Microscopy images covering the entire hippocampus were acquired and pre-processed to isolate the DG, removing the background and normalizing their intensity (Fig. 2A). To feed the images into the automated cell counting algorithm, splitting them into smaller tiles of 128×128 matrix size, followed by an interpolation to 1200×1200 matrix size was necessary. The tiles from all the GCaMP brain images were analyzed with a pre-trained CNN-based model in random order [23]. Part of the randomized tile pool is shown in Fig. 2B. The output of the model is a heatmap representing the probability of an object to correspond to a cell. When the probability exceeds 0.7, the object is considered a cell. This threshold value was previously shown to be within the optimal range for robust cell detection [23]. Subsequently, the detected cells from the tiles comprising one image were summed and all brain sections corresponding to the same brain were averaged (Fig. 2C). The brains that received TPS showed more than a two-fold increase in the c-Fos positive cell count compared to the sham-treated brains. The study also incorporated other strains to verify c-Fos levels in the DG following stimulation. Specifically, CD1 (N = 6) and 5xFAD (N = 6) animals (4 stimulated and 2 sham for each strain) were processed and analyzed similarly to the GCaMP + mice. Quantitative analysis showed a significant increase, based on unpaired Student’s t-test, on the order of 59.7 % (p = 0.0422) and 61.5 % (p = 0.0435) for the CD1 and 5xFAD brains, respectively (Fig. S1). Blinded histological analysis further confirmed consistent c-Fos upregulation across TPS-treated groups. Despite limited group numbers in these initial validation studies, the large effect size observed demonstrates a clear signal over noise.

### 3.3. TPS rapidly and transiently modulates brain network circuitry

Alterations in FC across brain networks have been observed in various neurological disorders, including AD [28], and they may serve as a biomarker to facilitate the assessment of disease progression and evaluate responses to potential treatments. We assessed the immediate impact of TPS stimulation on whole-brain FC with resting-state fMRI experiments conducted in WT mice (B6129SF2/J, N = 6) and AD (3xTg-AD, N = 6). The experimental procedure consisted of a 15-min baseline scan followed by two identical TPS stimulation sequences (TPS 1 and TPS 2; see ‘TPS Stimulation’ and ‘In vivo MRI’ subsections in Materials and Methods) interleaved with 15-min post-stimulation acquisitions (Post-TPS 1 and Post-TPS 2) (Fig. 3A). To resolve the temporal dynamics of TPS-induced FC changes, each scan was further segmented into 5-min intervals.

At the group level (N = 12), the baseline FC exhibited the anticipated patterns with robust intra-network connectivity across regions (see Table S1 for a complete list) (Fig. 3B). Immediately following TPS, a rapid reorganization of network synchrony was observed. The most pronounced alterations were detected in the hypothalamus, midbrain, basal ganglia, and hippocampus (Fig. 3C and D). After TPS 1, the strongest connectivity changes occurred during the initial 5-min post-stimulation interval, with most metrics reverting to baseline within 10 min (Fig. 3C). TPS 2 produced a similar pattern, although the robust alterations during the first 5 min were followed by attenuated changes that persisted into the subsequent 5–10 min window before returning to baseline (Fig. 3D).

### 3.4. TPS differentially alters FC across the limbic system

On a regional level, TPS elicited robust FC changes across diverse brain areas integral to memory, arousal, and emotion (Fig. 3E–F, Fig. S2). The hypothalamus emerged as a hotspot, exhibiting significant connectivity alterations across its subregions. Notably, the anterior hypothalamic area exhibited the most pronounced alterations, accompanied by significant changes in the periventricular hypothalamus, ventromedial hypothalamic nucleus, posterior hypothalamic nucleus, and paraventricular hypothalamic nucleus. Specifically, the anterior hypothalamic area showed increased FC with hippocampal regions (CA1, CA2, CA3, and entorhinal cortex), while similar enhancements were observed between the hypothalamus and other key limbic structures such as the lateral amygdalar nucleus, lateral septal nucleus, and striatum (Fig. 3E–G). In contrast, a reduction in FC between the hypothalamus and other regions including the midbrain reticular nucleus, lateral dorsal thalamus, and anterior pretectal nucleus (APN) was observed following TPS stimulation. Moreover, TPS augmented connectivity within the hypothalamic and hippocampal circuits. Intriguingly, supplementary network alterations were also observed. The APN, which plays a critical role in mediating the pupillary light reflex, displayed decreased FC with several key regions including the hypothalamus, striatum, amygdala, and lateral septal nucleus (Fig. 3E, Fig. S3).

Importantly, the region-wise FC changes induced by TPS were consistent across both WT and AD animals, exhibiting highly similar patterns and magnitudes of connectivity alterations (Fig. 3H). Moreover, the FC changes observed in each strain were significantly greater than those in the sham group (N = 4; 2 B6129SF2/J and 2 3xTg-AD, p < 0.05 for all region pairs; Student’s t-test; see Table S2 for detailed statistical measures) (Fig. 3H, Fig. S4).

## 4. Discussion

The primary purpose of this study has been exploring the neuronal activity and brain circuit responses to transcranial pulse stimulation, driving the cerebrovascular changes observed in our previous work [19]. Real-time FL imaging of the fluorophore sensor GCaMP with excitation wavelength at 488 nm served as a proxy of neuronal activity. Quantification of the relative FL signal changes (ΔF/F_0_) showed a significant increase in calcium activity, particularly during high-intensity stimulation, with a subsequent return to baseline. The excitatory effect of neuromodulation has previously been reported to originate from US mechanical forces without temperature increase or cavitational events [29]. The observed neuronal activity thus supports the anticipated strong neuromodulatory effects of TPS.

To further assess the spatial distribution of activated neurons, immunohistochemical analysis was conducted against the c-Fos protein. Automated c-Fos quantification of coronal brain sections, acquired with confocal microscopy, showed increased c-Fos presence in the hippocampal brain structure, compared to its inherent levels. c-Fos upregulation is associated with membrane depolarization and glial cell activation through the pathways of proliferation, differentiation, plasticity, and other cellular conditions related to the brain microenvironment [30]. Signaling pathways associated with c-Fos expression are known to be linked to calcium influx into neurons. Specifically, calcium ions enter neurons through voltage-gated calcium channels activated by action potentials, bind to calmodulin, and subsequently activate both calcium/calmodulin-dependent protein kinase (CaMK) II and cAMP-response element binding protein (CREB) [31]. Hence, data from FL imaging of the GCaMP mice and c-Fos activation adhere to such a mechanism.

Fluctuations in the brain circuitry were detected by resting state fMRI immediately following TPS within a duration of less than 10 min. The limbic system was upregulated, assessed by the increased FC from the hypothalamus to the hippocampus, the hypothalamus to the amygdala, and the hypothalamus to the entorhinal cortex. Given that the focal region of the clinical handpiece covers majority of the murine brain, these subcortical and hippocampal regions were encompassed within the stimulated volume. Temporal lobe deficits align with language and semantic deficits and are associated with depleted interhemispheric coherence in AD patients [32]. Temporal lobe functionality, including brain regions associated with memory formation, learning networks and memory formations, was restored following electrical stimulation indicated by synaptic remodeling, hinting a modified connectivity and plasticity [33]. Alterations in the APN indicate potential impacts on the processing of visual reflexive behaviors, aligning with previous observations of US-induced pupil dilation [34]. Collectively, this points to broader neuromodulatory effects of TPS with its influence extending to diverse aspects of brain function beyond traditional limbic networks.

Consistent with this broader effect, TPS also reorganized connectivity across subcortical circuits, including the basal ganglia and midbrain. These regions are essential hubs for motor as well as cognitive integration, whilst their modulation further supports the view that TPS exerts network-level effects across deep brain structures. Importantly, this aligns with recent clinical observations in Parkinson’s disease, where a single TPS session targeting the motor cortex was reported to alleviate resting tremor, pointing to functional engagement of motor circuitry [35]. Taken together, our preclinical evidence of subcortical network engagement provides a plausible mechanistic substrate for these early clinical findings, thus highlighting TPS as a promising neuromodulatory approach across both cognitive and motor domains.

The brain excitation effects shown herein are consistent with previous neuromodulation studies providing evidence of the mechanosensitive ion channel response to pulsed US waves [5–7,13–15,29]. Low-energy shock wave application overcomes the limitation of optimizing the safe parametric window of neuromodulation, with long pulse trains carrying higher risks of inducing thermal and cavitational effects [15]. The stimulation of calcium influx, indicative of neuronal activation, supported by the increase in c-Fos activity and the subsequent FC within the limbic system, suggest a plausible mechanism of TPS action, concurrent with existing literature from the neuromodulation field. Importantly, spreading depolarization waves were only observed at × 5 the intensities used for AD therapy in clinical settings, whereas clear calcium transients and c-Fos upregulation were also induced at clinically relevant TPS energy levels. These findings indicate that controlled induction of neuronal activity, rather than spreading depolarization, is the likely mechanism underlying the reported therapeutic benefits of TPS. This is consistent with other neuromodulation approaches, such as deep brain stimulation or transcranial magnetic stimulation, where modulation of circuit activity is achieved without triggering pathological depolarization.

US neuromodulation is increasingly being recognized as a promising therapeutic approach that alters neuronal activity to restore or modify brain function. In the context of AD and other neurodegenerative diseases, neuromodulation can target brain circuits involved in cognitive, motor, and emotional processes, which are disrupted under these conditions. The beneficial effects of TPS observed in AD [16,18,36–40], depression [41–43] and other conditions can be partially attributed to its neuromodulatory capabilities. The preclinical imaging platform employed in this study offers a powerful means to optimize stimulation parameters by simultaneously monitoring functional efficacy and safety within the same preparation. This is essential for identifying parameter ranges that induce robust neuronal and network responses while avoiding tissue damage or cavitation. At the same time, it is crucial to accurately adapt the per-pulse energy to clinical scenarios, as the thick human skulls manifest much stronger acoustic attenuation compared to the thin murine skulls [44]. In this regard, the 0.05 mJ/mm^2^ condition used here was selected to approximate the effective 0.25 mJ/mm^2^ clinical dose, while the higher 0.25 mJ/mm^2^ setting was included to probe the upper bound of neuromodulatory effects. Importantly, TPS applied at the present parameters has been shown to be safe in mice, with no evidence of blood–brain barrier opening, cavitation, or structural damage [19]. This favorable safety profile is a key advantage of TPS over other ultrasound-based neuromodulation techniques, further reinforcing its translational potential.

In conclusion, this study successfully demonstrates that TPS induces neuronal activity and associated brain circuit responses, as evidenced by the observed calcium influxes, c-Fos upregulation, and changes in FC within the limbic system. These findings support the hypothesis that the mechanical forces generated by TPS are sufficient to modulate neuronal activity and alter brain circuitry without causing tissue heating, cavitational or other detrimental effects on the brain. The results further underscore the potential of TPS as a non-invasive neuromodulatory technique that can promote neuronal activation and modulate brain function, offering promise for therapeutic applications in neurodegenerative diseases and other conditions.

## Supplementary Material

1

2

3

4

5

## Data Availability

All data are available in the main text or the supplementary materials.

## Appendix A. Supplementary data

Supplementary data to this article can be found online at https://doi.org/10.1016/j.brs.2025.09.021.
