# Supplementary material for: Transcranial pulse stimulation modulates neuronal activity and functional network dynamics
Source: Brain Stimul. Author manuscript; Available in PMC 2026 Jul 10. (PMC13352088; doi:10.1016/j.brs.2025.09.021)
Supplement: 1 [file NIHMS2183818-supplement-1.docx]

**Supplementary Material**

Transcranial pulse stimulation modulates neuronal activity and

functional network dynamics

Maria Eleni Karakatsani^1,2,§^, Irmak Gezginer^1,2,§^, Daniil Nozdriukhin^1,2^, Savannah Tiemann^1,2^, Hikari A. I. Yoshihara^1,2^, Rafael Storz^4^, Markus Belau^4^, Ruiqing Ni^1,2,3^, Xosé Luís Deán-Ben^1,2,*^, and Daniel Razansky^1,2,*^

^1^Institute for Biomedical Engineering and Institute of Pharmacology and Toxicology, Faculty of Medicine, University of Zurich, Winterthurerstrasse 190, CH-8057 Zurich, Switzerland

^2^Institute for Biomedical Engineering, Department of Information Technology and Electrical Engineering, ETH Zurich, Wolfgang-Pauli-Strasse 27, CH-8093 Zurich, Switzerland

^3^Institute for Regenerative Medicine, Wagistrasse 12, 9^th^ floor, CH-8952 Zurich, Switzerland

^4^Storz Medical AG, Lohstampfestrasse 8, CH-8274Tagerwilen, Switzerland

^§^These authors have contributed equally

^*^Correspondence to: [xl.deanben@pharma.uzh.ch](mailto:xl.deanben@pharma.uzh.ch), [daniel.razansky@uzh.ch](mailto:daniel.razansky@uzh.ch)

**
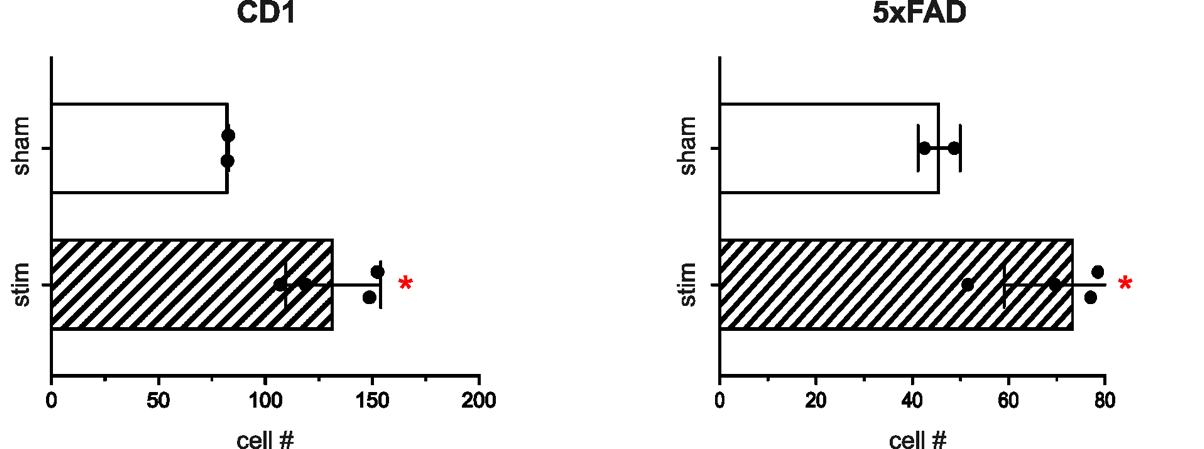
**

**Fig. S1.** Quantitative analysis of the c-Fos positive cells in the CD1 and 5xFAD brains (4 stimulated and 2 sham for each strain). A significant increase, based on unpaired Student's t-test, is shown on the order of 59.7% (p=0.0422) and 61.5% (p=0.0435) for the CD1 and 5xFAD brains respectively.

**
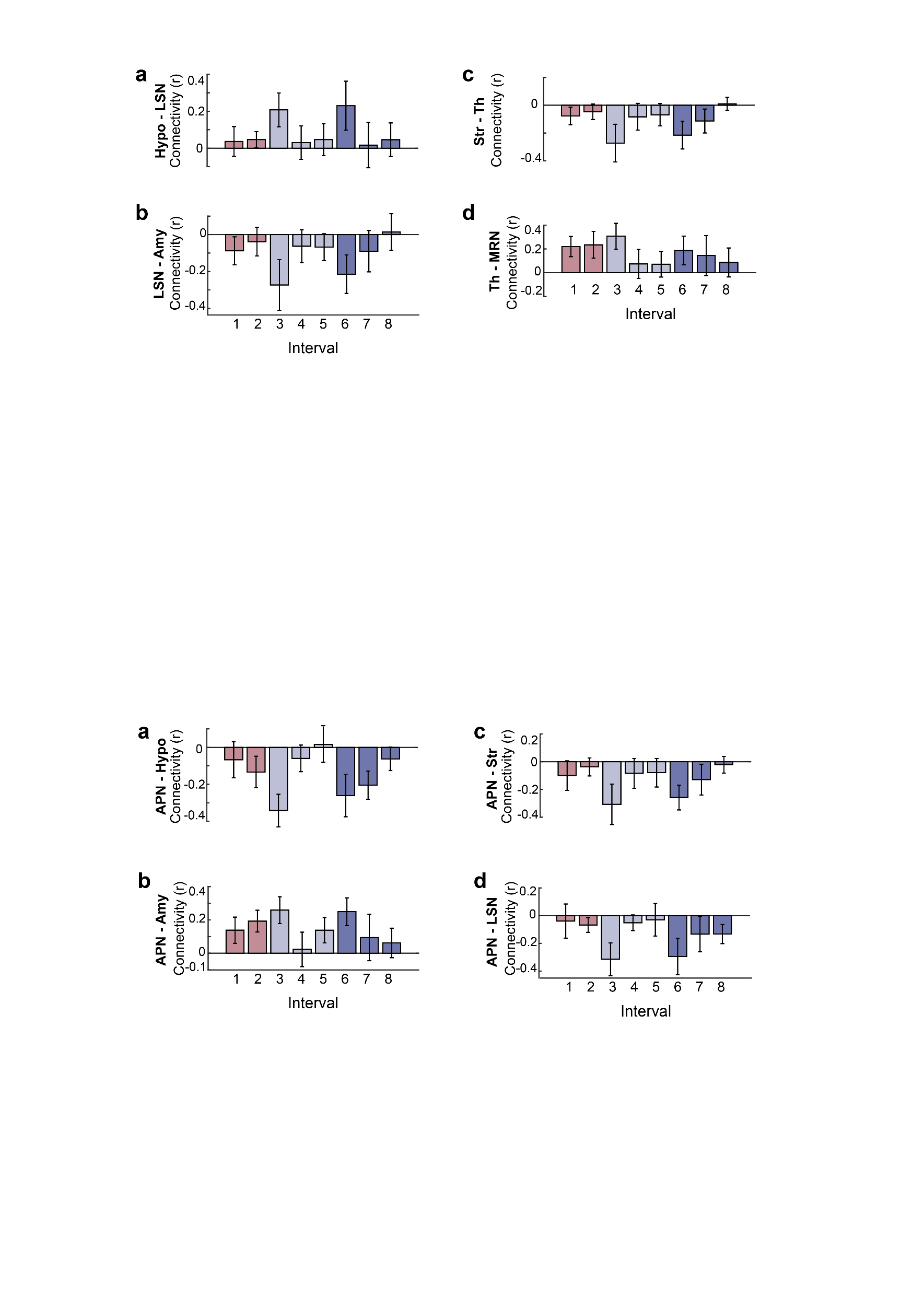
**

**A**

**C**

**B**

**D**

**Fig. S2.** Group-averaged FC values (mean ± SEM) for each time interval, illustrating the dynamics of region-wise connectivities. MRN, midbrain reticular nucleus; Hypo, anterior hypothalamic area; LSN, lateral septal nucleus; Amy, lateral amygdalar nucleus; Th, lateral dorsal thalamus; Str, Striatum.

**
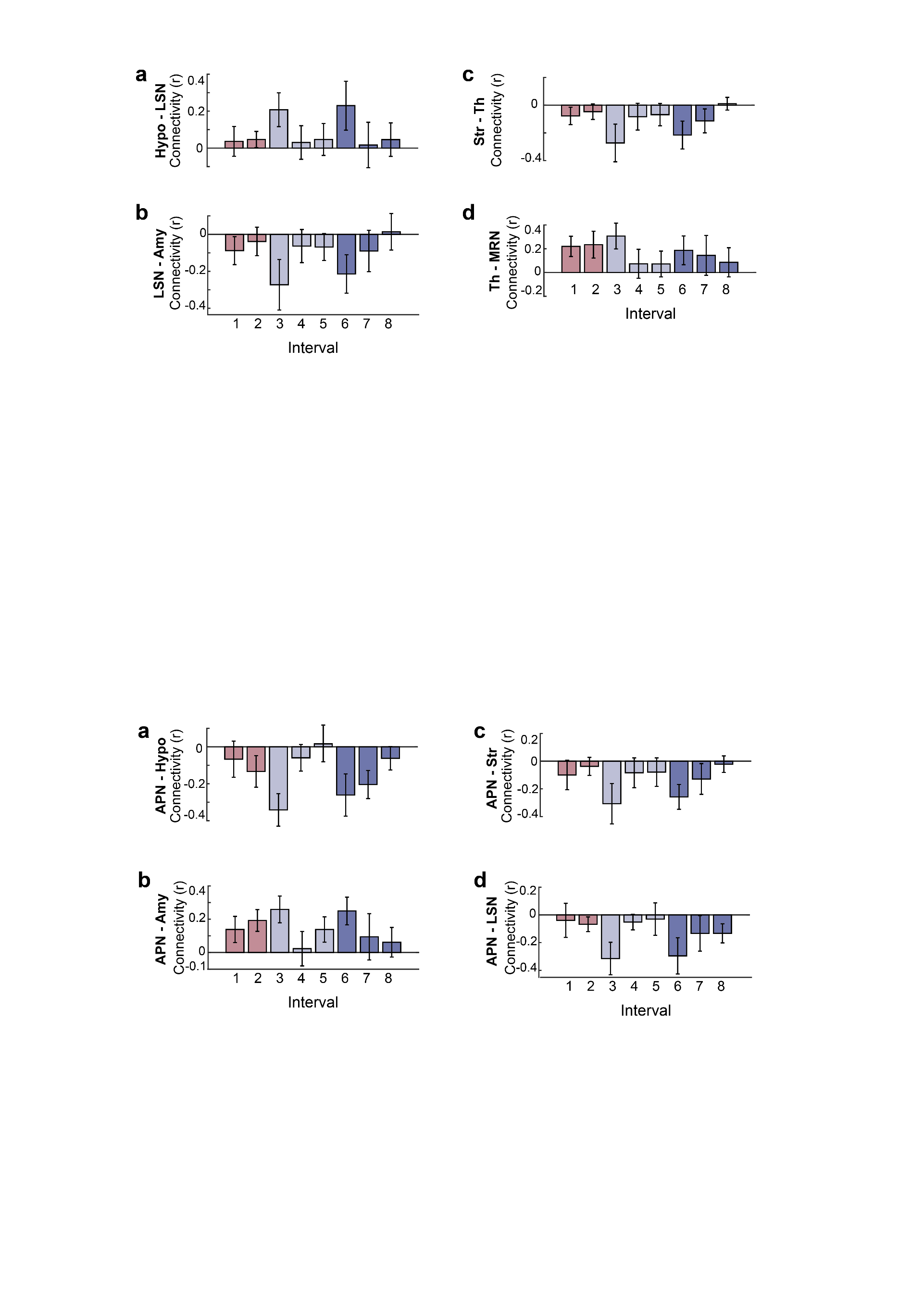
**

**C**

**B**

**A**

**Fig. S3.** Group-averaged FC changes (mean ± SEM) of anterior pretectal nucleus (APN) to various brain regions. Hypo, anterior hypothalamic area; LSN, lateral septal nucleus; Amy, lateral amygdalar nucleus; Str, Striatum.

**D**

**
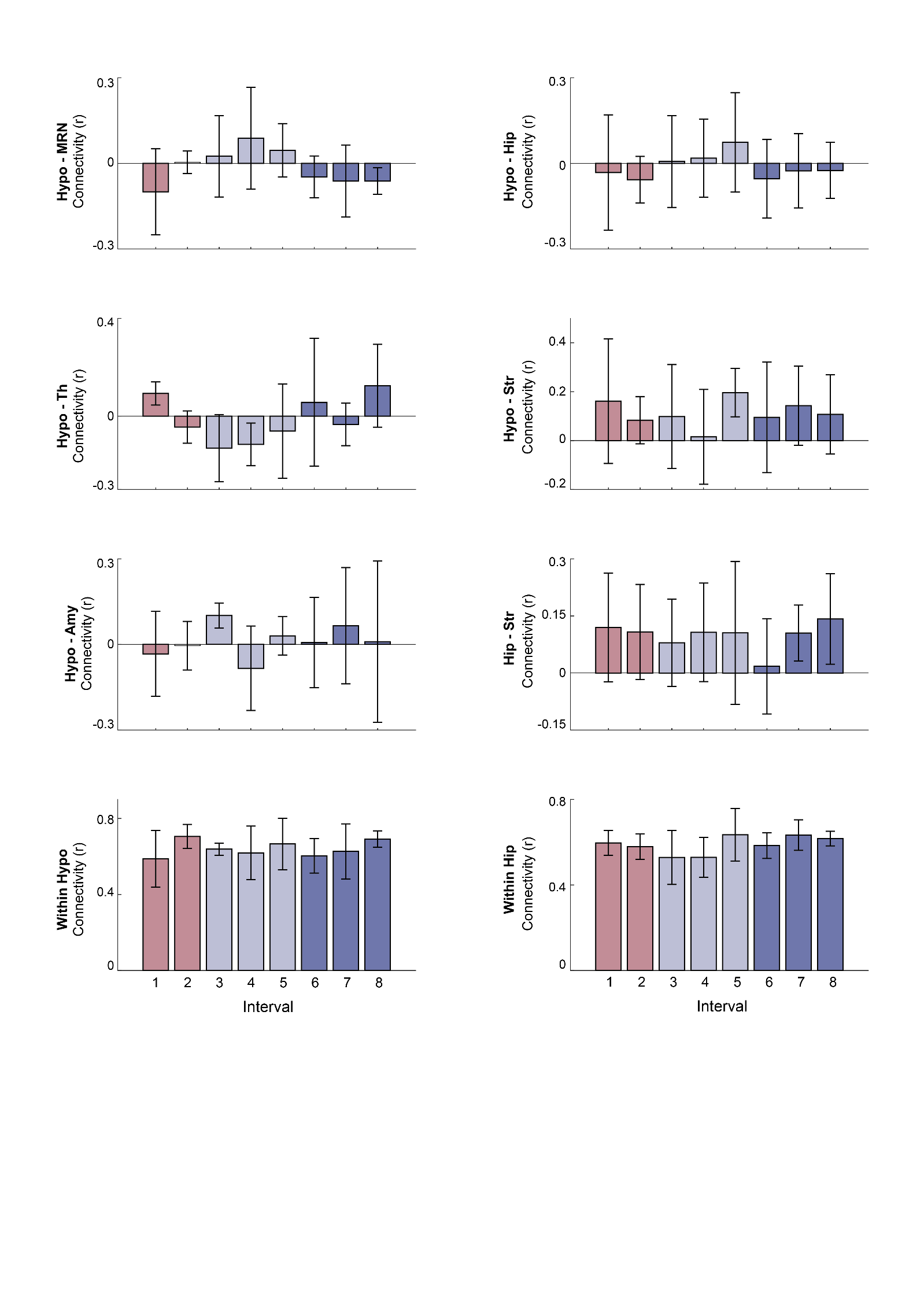
**

**Fig. S4.** Group-averaged (n = 4) FC changes (mean ± SEM) of brain region-pairs in response to sham experiment. MRN, midbrain reticular nucleus; Hypo, anterior hypothalamic area; LSN, lateral septal nucleus; Hip, hippocampus; Amy, lateral amygdalar nucleus; Th, lateral dorsal thalamus; Str, Striatum; APN, anterior pretectal nucleus.

**Table S1.** List of regions considered in fMRI analysis.

| **Region Name** | **Region Name (continued)** |
| --- | --- |
| Primary motor area | Field CA1 |
| Secondary motor area | Field CA2 |
| Primary somatosensory area, nose | Field CA3 |
| Primary somatosensory area, barrel field | Dentate gyrus |
| Primary somatosensory area, lower limb | Entorhinal area, lateral part |
| Primary somatosensory area, mouth | Entorhinal area, medial part |
| Primary somatosensory area, upper limb | Subiculum |
| Primary somatosensory area, trunk | Endopiriform nucleus |
| Supplemental somatosensory area | Lateral amygdalar nucleus |
| Gustatory areas | Striatum |
| Visceral area | Caudoputamen |
| Dorsal auditory area | Nucleus accumbens |
| Primary auditory area | Lateral septal nucleus |
| Posterior auditory area | Central amygdalar nucleus |
| Ventral auditory area | Globus pallidus |
| Anterolateral visual area | Bed nuclei of the stria terminalis |
| Anteromedial visual area | Thalamus |
| Lateral visual area | Ventral posterior complex of the thalamus |
| Primary visual area | Lateral group of the dorsal thalamus |
| Posterolateral visual area | Anterior group of the dorsal thalamus |
| Posteromedial visual area | Medial group of the dorsal thalamus |
| Postrhinal area | Hypothalamus |
| Anterior cingulate area, dorsal part | Periventricular zone of hypothalamus |
| Anterior cingulate area, ventral part | Anterior hypothalamic area |
| Prelimbic area | Ventromedial hypothalamic nucleus |
| Infralimbic area | Posterior hypothalamic nucleus |
| Orbital area, lateral part | Lateral hypothalamic area |
| Orbital area, medial part | Midbrain |
| Orbital area, ventrolateral part | Superior colliculus, sensory related |
| Agranular insular area, dorsal part | Inferior colliculus |
| Agranular insular area, posterior part | Substantia nigra, reticular part |
| Agranular insular area, ventral part | Ventral tegmental area |
| Retrosplenial area, lateral agranular part | Midbrain reticular nucleus |
| Retrosplenial area, dorsal part | Anterior pretectal nucleus |
| Retrosplenial area, ventral part | Cuneiform nucleus |
| Anterior area | Red nucleus |
| Rostrolateral visual area | Pedunculopontine nucleus |
| Temporal association areas | Pons |
| Perirhinal area | Nucleus of the lateral lemniscus |
| Ectorhinal area | Principal sensory nucleus of the trigeminal |
| Anterior olfactory nucleus | Parabrachial nucleus |
| Piriform area | Pontine reticular nucleus |
| Cortical amygdalar area | Superior central nucleus raphe |
| Hippocampal formation |  |

**Table S2**. Statistical measures related to region-wise functional connectivity (FC) changes induced by TPS across stimulated wild-type (WT), stimulated Alzheimer’s (AD), and sham animals. The following information follows Figure 3H in the main text.

| Region pair | FC change (Δr, mean ± SEM) | | | p-value (two-sample t-test) | | |
| --- | --- | --- | --- | --- | --- | --- |
|  | WT | AD | Sham | WT vs AD | WT vs Sham | AD vs Sham |
| Hypo - MRN | -0.172 ± 0.061 | -0.155 ± 0.050 | 0.062 ± 0.054 | 0.831 | 0.015 | 0.010 |
| Hypo - Th | -0.200 ± 0.086 | -0.207 ± 0.075 | 0.092 ± 0.084 | 0.951 | 0.033 | 0.018 |
| Hypo – Amy | 0.144 ± 0.050 | 0.285 ± 0.065 | -0.067 ± 0.078 | 0.102 | 0.027 | 0.003 |
| Within Hypo | 0.135 ± 0.024 | 0.116 ± 0.025 | -0.065 ± 0.033 | 0.591 | < 0.001 | <0.001 |
| Hypo – Hip | 0.143 ± 0.058 | 0.204 ± 0.056 | -0.032 ± 0.077 | 0.459 | 0.050 | 0.021 |
| Hypo – Str | 0.199 ± 0.047 | 0.119 ± 0.049 | -0.153 ± 0.048 | 0.251 | < 0.001 | 0.001 |
| Hip – Str | 0.156 ± 0.062 | 0.097 ± 0.060 | -0.085 ± 0.074 | 0.502 | 0.023 | 0.042 |
| Within Hip | 0.105 ± 0.042 | 0.088 ± 0.038 | -0.051 ± 0.052 | 0.761 | 0.031 | 0.040 |
